# Two polymorphs of [Rh(μ-I)(COD)]_2_

**DOI:** 10.1107/S205698902100743X

**Published:** 2021-07-27

**Authors:** David R. Ullery, Curtis E. Moore, Christine M. Thomas

**Affiliations:** a 100 W. 18<sup>th</sup> Ave., Department of Chemistry and Biochemistry, The Ohio State University, Columbus, OH 43210, USA

**Keywords:** crystal structure, polymorph, rhodium, dimer

## Abstract

The solid-state structure of di-μ-iodido-bis­{[(1,2,5,6-η)-cyclo­octa-1,4-diene]rhodium(I)}, [Rh_2_I_2_(C_8_H_12_)_2_] or [Rh(μ-I)(COD)]_2_, was determined from two crystals with different morphologies, which were found to correspond to two polymorphs containing Rh dimers with significantly different mol­ecular structures. Both polymorphs are monoclinic and the [Rh(μ-I)(COD)]_2_ mol­ecules in each case possess C_2_ *v* symmetry. However, the core geometry of the butterfly-shaped Rh_2_I_2_ core differs substanti­ally. In the *C*2/*c* polymorph, the core geometry of [Rh(μ-I)(COD)]_2_^B^ is bent, with a hinge angle of 96.13 (8)° and a Rh⋯Rh distance of 2.9612 (11) Å. The *P*2_1_/*c* polymorph features a more planar [Rh(μ-I)(COD)]_2_^P^ core geometry, with a hinge angle of 145.69 (9)° and a Rh⋯Rh distance of 3.7646 (5) Å.

## Abstract 2

[Rh(μ-I)(COD)]_2_ was found to crystallize as two different polymorphs in which the Rh dimer adopts either bent or planar geometries with respect to the Rh_2_I_2_ core.

## Chemical context   

Compounds of the type [*M*(μ-*X*)(COD)]_2_, (*M* = Ir, Rh, *X* = Cl, Br, I) are ubiquitous synthons for Rh and Ir catalysts. As a representative example, [Rh(COD)(DPEphos)]BF_4_ catalyzes the hydro­amination of vinyl­arenes with anti-Markovnikov selectivity, and is prepared *via* the reaction of [Rh(μ-Cl)(COD)]_2_ with two equivalents of AgBF_4_ and two equivalents of DPEphos (Utsunomiya *et al.*, 2003 ▸). Within the series [*M*(μ-*X*)(COD)]_2_ (*M* = Ir, Rh; *X* = Cl, Br, I), all compounds have been structurally characterized with the notable exception of [Rh(μ-I)(COD)]_2_ (De Ridder & Imhoff, 1994 ▸; Pettinari *et al.*, 2002 ▸; Cotton *et al.*, 1986 ▸; Yamagata *et al.*, 2007*a* ▸,*b* ▸). Thus, crystallographic characterization of the title compound was pursued to complete the series.
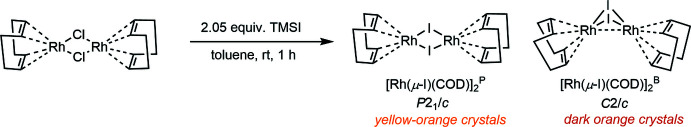


## Structural commentary   

**Figure 1:**
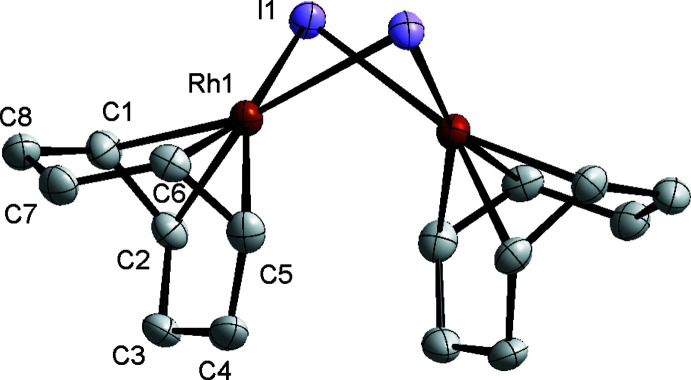
Bent structure of [Rh(μ-I)(COD)]_2_^B^ within the monoclinic *C*2/*c* polymorph. Independent atoms are labelled, while the other half of the molecule is symmetry-generated through a twofold rotation axis. Ellipsoids are drawn at the 50% probability level. Hydrogen atoms are removed for clarity.

Crystallization of [Rh(μ-I)(COD)]_2_ in toluene at 236 K produced two types of crystals with different colors and morphologies, namely dark-orange and yellow–orange blocks. A representative crystal of each type was subjected to single crystal X-ray diffraction, revealing two different polymorphs of [Rh(μ-I)(COD)]_2_ containing dimers with significantly different structural features. X-ray diffraction of the dark-orange crystals revealed that [Rh(μ-I)(COD)]_2_ crystallized in the monoclinic *C*2/*c* space group in this polymorph. The mol­ecular structure of [Rh(μ-I)(COD)]_2_^B^ exhibited a C_2_ *v*-symmetric geometry featuring a bent Rh_2_I_2_ diamond core (Fig. 1 ▸). The hinge angle, defined as the angle between the inter­secting planes that contain the iodide ligands and each rhodium, is 96.13 (8)° and the Rh⋯Rh distance is 2.9612 (11) Å. [Rh(μ-I)(COD)]_2_^B^ exhibits a geometry significantly different from that of [Rh(μ-Cl)(COD)]_2_ and [Rh(μ-Br)(COD)]_2_, which both exhibit more planar Rh_2_ *X*_2_ cores with hinge angles of 169.08 (6) and 148.74 (7)° and Rh⋯Rh distances of 3.5169 (6) and 3.5648 (14) Å, respectively (De Ridder & Imhoff, 1994 ▸; Pettinari *et al.*, 2002 ▸). However, the hinge angle of [Rh(μ-I)(COD)]_2_^B^ is similar to that of [Ir(μ-I)(COD)]_2_ [95.26 (1)°; Yamagata *et al.*, 2007*b* ▸].

**Figure 2:**
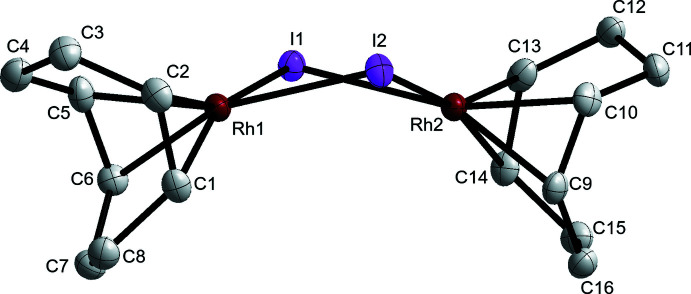
Planar structure of [Rh(μ-I)(COD)]_2_^P^ within the monoclinic *P*2_1_/*c* polymorph. Ellipsoids are drawn at the 50% probability level. Hydrogen atoms are removed for clarity.

X-ray diffraction of the yellow–orange crystals revealed a different polymorph of [Rh(μ-I)(COD)]_2_, this time crystallizing in the monoclinic *P*2_1_/*c* space group. The geometry of the dirhodium dimer in the *P*2_1_/*c* polymorph [Rh(μ-I)(COD)]_2_^P^ differs significantly from the *C*2/*c* polymorph [Rh(μ-I)(COD)]_2_^B^. The Rh_2_I_2_ core geometry of [Rh(μ-I)(COD)]_2_^P^ is more planar, with a hinge angle of 145.69 (9)° and a Rh⋯Rh distance of 3.7646 (5) Å (Fig. 2 ▸). The mol­ecular structure of [Rh(μ-I)(COD)]_2_^P^ is therefore similar to [Rh(μ-Cl)(COD)]_2_ and [Rh(μ-Br)(COD)]_2_ (De Ridder & Imhoff, 1994 ▸; Pettinari *et al.*, 2002 ▸).

A previous theoretical study found relatively small energetic differences (< 10 kcal mol^−1^) between planar and bent [Rh(μ-*X*)(*L*)_2_]_2_ geometries (Aullón *et al.*, 1998 ▸). By analyzing the donor–acceptor inter­actions between *d_z_* ^2^ and *p_z_* orbitals of the two metal atoms, it was determined that the stability of bent morphologies increases as the electronegativity of the bridging ligand decreases. The degree of bending was predicted to increase in the order Cl < Br < I, consistent with the observation of a bent structure for [Rh(μ-I)(COD)]_2_^B^ but not [Rh(μ-Cl)(COD)]_2_ and [Rh(μ-Br)(COD)]_2_ (Aullón *et al.*, 1998 ▸). Moreover, Aullón and coworkers predicted the bent form to be more stable for Ir than for Rh, in line with the exclusive observation of a bent geometry for [Ir(μ-I)(COD)]_2_, but the possibility of both planar and bent forms for [Rh(μ-I)(COD)]_2_.

There is no meaningful difference between the two independent Rh—I distances in [Rh(μ-I)(COD)]_2_^B^ [2.7072 (7) and 2.6975 (7) Å]. The four Rh—I distances in [Rh(μ-I)(COD)]_2_^P^ are slightly less symmetric: the bonds between I2 and the two Rh centers [2.6833 (4) and 2.6738 (4) Å] are slightly shorter than those associated with I1 [2.6998 (4) and 2.7061 (4) Å]. Similarly, the Rh—C distances in [Rh(μ-I)(COD)]_2_^B^ are more symmetric, ranging from 2.115 (6) to 2.122 (6) Å, while the Rh—C distances in [Rh(μ-I)(COD)]_2_^P^ range from 2.117 (4) to 2.131 (4) Å. The average Rh—C distance in the bent and planar structures are similar to the average Rh—C distances reported for the [Rh(μ-Cl)(COD)]_2_ and [Rh(μ-Br)(COD)]_2_ analogues (De Ridder & Imhoff, 1994 ▸; Pettinari *et al.*, 2002 ▸), with all four compounds having an average Rh—C distance of 2.12 Å. As expected based on the inherent differences in covalent radii, the average Rh—I distances in [Rh(μ-I)(COD)]_2_^B^ and [Rh(μ-I)(COD)]_2_^P^ (2.70 and 2.69 Å, respectively) are considerably longer than the average Rh—Br and Rh—Cl distances in [Rh(μ-Br)(COD)]_2_ and [Rh(μ-Cl)(COD)]_2_ (2.54 and 2.38 Å, respectively).

## Supra­molecular features   

**Figure 3:**
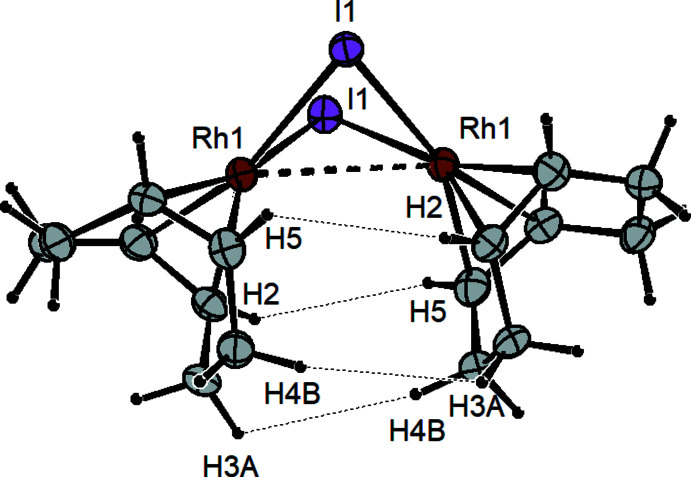
Diagram of [Rh(μ-I)(COD)]_2_^B^ showing weak intra­molecular C—H⋯H—C dispersion inter­actions between the two COD mol­ecules on the two Rh centers.

**Figure 4:**
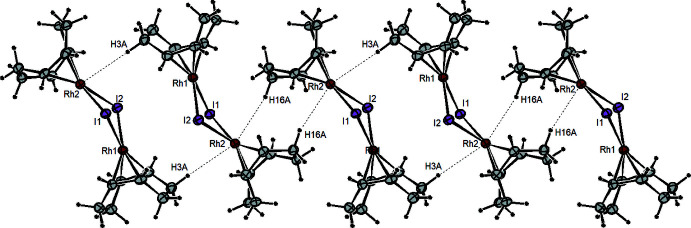
Diagram of [Rh(μ-I)(COD)]_2_^P^ showing weak inter­molecular C—H⋯Rh inter­actions between adjacent mol­ecules.

The structural differences between the dimers in the two polymorphs of [Rh(μ-I)(COD)]_2_ are attributed to differences in crystal packing and weak inter­atomic forces. The bent and planar geometries are likely similar in energy. Stabilization of the bent geometry in [Rh(μ-I)(COD)]_2_^B^ arises from intra­molecular dispersion forces between the C—H bonds of the cyclo­octa­diene ligands on the two Rh centers within each mol­ecule. Indeed there are four close C—H⋯H—C contacts (H2⋯H5 = 2.64 Å; H3*A*⋯H4*B* = 2.66 Å) between the alkene and methyl­ene hydrogen atoms made possible by the bent geometry (Fig. 3 ▸). In the case of [Rh(μ-I)(COD)]_2_^P^, no such intra­molecular C—H⋯H—C inter­actions are present. The shortest inter­molecuar inter­actions in [Rh(μ-I)(COD)]_2_^P^ are two Rh⋯H—C contacts in the apical positions of Rh2 (Rh2⋯H16*A*(1 − *x*, 1 − *y*, −*z*) = 2.67 Å; Rh2⋯H3*A*(2 − *x*, 1 − *y*, 1 − *z*) = 2.93 Å) that could, at best, be labeled as weak inter­molecular agostic inter­actions (Fig. 4 ▸). Consistent with the inter­molecular inter­actions in the planar structure, the *P*2_1_/*c* has a higher density (2.621 g cm^−3^) than the bent *C*2/*c* polymorph (2.597 g cm^−3^), indicative of tighter crystal packing.

## Synthesis and crystallization   

[Rh(μ-I)(COD)]_2_ was prepared according to the procedure described by J. A. Hlina *et al.* (2017 ▸). Under a nitro­gen atmosphere, [Rh(μ-Cl)(COD)]_2_ (312.0 mg, 0.6323 mmol) was added to toluene (5 mL) and tri­methyl­silyl iodide (184.6 µL, 1.297 mmol). The reaction mixture turned dark red and rust-colored crystals precipitated from the solution. The solid was isolated, washed with hexa­nes and dried *in vacuo* to yield the final product [Rh(μ-I)(COD)]_2_ as a red–brown crystalline solid (358.5 mg, 84%). X-ray quality crystals were grown from a concentrated solution of toluene at 236 K, resulting in crystals with two different morphologies. ^1^H NMR (C_6_D_6_): 1.15–1.28 (*m*, 8H, –C*H*H–), 1.90–2.05 (*m*, 8H, –CH*H*–), 4.62–4.70 (*m*, 8H, =C*H*). A single species was observed by ^1^H NMR spectroscopy and the ^1^H NMR spectrum did not contain any broad features indicative of dynamic behavior or inter­conversion between the two isomers in solution.

## Refinement   

Crystal data, data collection and structure refinement details are summarized in Table 1 ▸. Crystals were mounted on MiTeGen Micromounts with Paratone 24EX oil. Data were collected in a nitro­gen gas stream at 100 (2) K using φ and ω scans. Solution by direct methods (*SHELXT*) produced a complete phasing model for refinement. All non-hydrogen atoms were refined anisotropically by full-matrix least-squares (*SHELXL2018*). All carbon-bonded methyl­ene hydrogen atoms were placed using a riding model. Their positions were constrained relative to their parent atom using the appropriate HFIX command in *SHELXL2018*. All carbon-bonded methine hydrogen atoms were located in the difference map. Their C—H distances were restrained to a target value of 1.00 (2) Å. For all H atoms, displacement parameter *U*_iso_(H) values were set to 1.2 times *U*_eq_(C).

## Supplementary Material

Crystal structure: contains datablock(s) global, B, P. DOI: 10.1107/S205698902100743X/zl5015sup1.cif

Structure factors: contains datablock(s) B. DOI: 10.1107/S205698902100743X/zl5015Bsup2.hkl

Structure factors: contains datablock(s) P. DOI: 10.1107/S205698902100743X/zl5015Psup3.hkl

CCDC references: 2097568, 2097567

Additional supporting information:  crystallographic information; 3D view; checkCIF report

## Figures and Tables

**Table 1:** Experimental details

	[Rh(μ-I)(COD)]_2_^B^	[Rh(μ-I)(COD)]_2_^P^
Crystal data		
Chemical formula	[Rh_2_I_2_(C_8_H_12_)_2_]	[Rh_2_I_2_(C_8_H_12_)_2_]
*M*_r_	675.97	675.97
Crystal system, space group	Monoclinic, *C*2/*c*	Monoclinic, *P*2_1_/*c*
Temperature (K)	100	100
*a*, *b*, *c* (Å)	12.3414 (16), 11.8176 (17), 11.9374 (15)	10.4505 (5), 19.390 (1), 8.6271 (4)
β (°)	96.690 (3)	101.523 (2)
*V* (Å^3^)	1729.2 (4)	1712.92 (14)
*Z*	4	4
Radiation type	Mo *K*α	Mo *K*α
μ (mm^−1^)	5.47	5.52
Crystal size (mm)	0.10 × 0.06 × 0.03	0.15 × 0.14 × 0.09

Data collection		
Diffractometer	Nonius Kappa APEXII	Nonius Kappa APEXII
Absorption correction	Multi-scan (*SADABS*; Krause *et al.*, 2015 ▸)	Multi-scan (*SADABS*; Krause *et al.*, 2015 ▸)
*T*_min_, *T*_max_	0.062, 0.093	0.057, 0.093
No. of measured, independent and observed [*I* > 2σ(*I*)] reflections	15175, 1775, 1320	38760, 3519, 2922
*R*_int_	0.080	0.051

Refinement		
*R*[*F*^2^ > 2σ(*F* ^2^)], *wR*(*F* ^2^), *S*	0.035, 0.079, 1.03	0.025, 0.055, 1.07
No. of reflections	1775	3519
No. of parameters	103	205
No. of restraints	4	8
H-atom treatment	H atoms treated by a mixture of independent and constrained refinement	H atoms treated by a mixture of independent and constrained refinement
Δρ_max_, Δρ_min_ (e Å^−3^)	1.36, −0.79	1.26, −0.67

Computer programs: *APEX3* and *SAINT* (Bruker, 2017 ▸), *SHELXT* (Sheldrick, 2015*a* ▸), *SHELXL* (Sheldrick, 2015*b* ▸) and *OLEX2* (Dolomanov *et al.*, 2009 ▸).

## supplementary crystallographic information

### Di-µ-iodido-bis{[(1,2,5,6-η)-cycloocta-1,4-diene]rhodium(I)} (B) Crystal data

**Table:**

[Rh_2_I_2_(C_8_H_12_)_2_]	*F*(000) = 1264
*M**_r_* = 675.97	*D*_x_ = 2.597 Mg m^−^^3^
Monoclinic, *C*2/*c*	Mo *K*α radiation, λ = 0.71073 Å
*a* = 12.3414 (16) Å	Cell parameters from 2574 reflections
*b* = 11.8176 (17) Å	θ = 2.8–23.3°
*c* = 11.9374 (15) Å	µ = 5.47 mm^−^^1^
β = 96.690 (3)°	*T* = 100 K
*V* = 1729.2 (4) Å^3^	Block, dark orange
*Z* = 4	0.10 × 0.06 × 0.03 mm

### Di-µ-iodido-bis{[(1,2,5,6-η)-cycloocta-1,4-diene]rhodium(I)} (B) Data collection

**Table:**

Nonius Kappa APEXII diffractometer	1775 independent reflections
Radiation source: sealed tube, fine-focus	1320 reflections with *I* > 2σ(*I*)
Graphite monochromator	*R*_int_ = 0.080
Detector resolution: 7.9 pixels mm^-1^	θ_max_ = 26.4°, θ_min_ = 2.4°
ω and φ scans	*h* = −15→15
Absorption correction: multi-scan (SADABS; Krause *et al.*, 2015)	*k* = −14→14
*T*_min_ = 0.062, *T*_max_ = 0.093	*l* = −14→14
15175 measured reflections	

### Di-µ-iodido-bis{[(1,2,5,6-η)-cycloocta-1,4-diene]rhodium(I)} (B) Refinement

**Table:**

Refinement on *F*^2^	Primary atom site location: dual
Least-squares matrix: full	Secondary atom site location: difference Fourier map
*R*[*F*^2^ > 2σ(*F*^2^)] = 0.035	Hydrogen site location: mixed
*wR*(*F*^2^) = 0.079	H atoms treated by a mixture of independent and constrained refinement
*S* = 1.03	*w* = 1/[σ^2^(*F*_o_^2^) + (0.0302*P*)^2^] where *P* = (*F*_o_^2^ + 2*F*_c_^2^)/3
1775 reflections	(Δ/σ)_max_ < 0.001
103 parameters	Δρ_max_ = 1.36 e Å^−^^3^
4 restraints	Δρ_min_ = −0.79 e Å^−^^3^

### Di-µ-iodido-bis{[(1,2,5,6-η)-cycloocta-1,4-diene]rhodium(I)} (B) Special details

**Table:**

Geometry. All esds (except the esd in the dihedral angle between two l.s. planes) are estimated using the full covariance matrix. The cell esds are taken into account individually in the estimation of esds in distances, angles and torsion angles; correlations between esds in cell parameters are only used when they are defined by crystal symmetry. An approximate (isotropic) treatment of cell esds is used for estimating esds involving l.s. planes.

### Di-µ-iodido-bis{[(1,2,5,6-η)-cycloocta-1,4-diene]rhodium(I)} (B) Fractional atomic coordinates and isotropic or equivalent isotropic displacement parameters (Å^2^)

**Table:**

	*x*	*y*	*z*	*U*_iso_*/*U*_eq_	
I1	0.52770 (3)	0.20354 (3)	0.40381 (4)	0.02475 (15)	
Rh1	0.38142 (4)	0.31608 (4)	0.25934 (4)	0.02137 (15)	
C1	0.2885 (6)	0.3639 (6)	0.3898 (6)	0.0274 (16)	
H1	0.309 (5)	0.316 (5)	0.458 (3)	0.033*	
C2	0.3652 (5)	0.4497 (6)	0.3749 (5)	0.0255 (15)	
H2	0.433 (3)	0.456 (5)	0.429 (4)	0.031*	
C3	0.3323 (6)	0.5616 (5)	0.3199 (6)	0.0294 (16)	
H3A	0.380935	0.621981	0.354264	0.035*	
H3B	0.256778	0.580087	0.333793	0.035*	
C4	0.3390 (6)	0.5579 (5)	0.1903 (6)	0.0312 (17)	
H4A	0.282371	0.608659	0.152182	0.037*	
H4B	0.410972	0.587248	0.175166	0.037*	
C5	0.3237 (5)	0.4422 (6)	0.1410 (6)	0.0273 (16)	
H5	0.357 (5)	0.418 (5)	0.074 (3)	0.033*	
C6	0.2378 (5)	0.3707 (5)	0.1589 (5)	0.0229 (15)	
H6	0.219 (5)	0.306 (3)	0.107 (4)	0.028*	
C7	0.1447 (5)	0.4044 (6)	0.2257 (5)	0.0295 (16)	
H7A	0.076675	0.366941	0.192462	0.035*	
H7B	0.133403	0.487216	0.219759	0.035*	
C8	0.1680 (5)	0.3714 (6)	0.3502 (6)	0.0286 (16)	
H8A	0.134470	0.428113	0.396580	0.034*	
H8B	0.133657	0.297304	0.361920	0.034*	

### Di-µ-iodido-bis{[(1,2,5,6-η)-cycloocta-1,4-diene]rhodium(I)} (B) Atomic displacement parameters (Å^2^)

**Table:**

	*U*^11^	*U*^22^	*U*^33^	*U*^12^	*U*^13^	*U*^23^
I1	0.0234 (3)	0.0275 (2)	0.0232 (3)	0.00135 (18)	0.00190 (18)	0.00455 (18)
Rh1	0.0203 (3)	0.0237 (3)	0.0200 (3)	0.0009 (2)	0.0018 (2)	0.0007 (2)
C1	0.031 (4)	0.029 (4)	0.024 (4)	0.000 (3)	0.007 (3)	−0.004 (3)
C2	0.028 (4)	0.027 (4)	0.022 (4)	0.004 (3)	0.003 (3)	−0.004 (3)
C3	0.030 (4)	0.020 (4)	0.038 (4)	0.005 (3)	0.006 (3)	−0.002 (3)
C4	0.028 (4)	0.024 (4)	0.042 (5)	0.003 (3)	0.006 (3)	0.008 (3)
C5	0.025 (4)	0.034 (4)	0.022 (4)	0.004 (3)	−0.001 (3)	0.004 (3)
C6	0.025 (4)	0.025 (4)	0.017 (4)	0.002 (3)	−0.006 (3)	−0.003 (3)
C7	0.024 (4)	0.029 (4)	0.034 (4)	0.005 (3)	−0.001 (3)	0.002 (3)
C8	0.029 (4)	0.026 (4)	0.031 (4)	0.002 (3)	0.006 (3)	0.005 (3)

### Di-µ-iodido-bis{[(1,2,5,6-η)-cycloocta-1,4-diene]rhodium(I)} (B) Geometric parameters (Å, º)

**Table:**

I1—Rh1	2.6975 (7)	C1—C8	1.509 (9)
I1—Rh1^i^	2.7072 (7)	C2—C3	1.510 (9)
Rh1—Rh1^i^	2.9612 (11)	C3—C4	1.560 (9)
Rh1—C1	2.115 (6)	C4—C5	1.492 (9)
Rh1—C2	2.122 (6)	C5—C6	1.392 (9)
Rh1—C5	2.119 (7)	C6—C7	1.526 (9)
Rh1—C6	2.121 (6)	C7—C8	1.532 (9)
C1—C2	1.413 (9)		
			
Rh1—I1—Rh1^i^	66.45 (2)	C6—Rh1—I1	164.61 (18)
I1—Rh1—I1^i^	85.13 (2)	C6—Rh1—Rh1^i^	136.51 (18)
I1^i^—Rh1—Rh1^i^	56.621 (18)	C6—Rh1—C2	90.3 (2)
I1—Rh1—Rh1^i^	56.933 (19)	C2—C1—Rh1	70.8 (4)
C1—Rh1—I1^i^	165.07 (19)	C2—C1—C8	124.6 (6)
C1—Rh1—I1	92.3 (2)	C8—C1—Rh1	112.6 (4)
C1—Rh1—Rh1^i^	132.91 (19)	C1—C2—Rh1	70.3 (4)
C1—Rh1—C2	39.0 (2)	C1—C2—C3	122.2 (6)
C1—Rh1—C5	97.7 (3)	C3—C2—Rh1	114.2 (5)
C1—Rh1—C6	81.1 (3)	C2—C3—C4	111.4 (5)
C2—Rh1—I1^i^	155.75 (18)	C5—C4—C3	113.4 (5)
C2—Rh1—I1	93.32 (18)	C4—C5—Rh1	111.4 (5)
C2—Rh1—Rh1^i^	102.57 (18)	C6—C5—Rh1	70.9 (4)
C5—Rh1—I1	157.07 (18)	C6—C5—C4	124.1 (6)
C5—Rh1—I1^i^	90.08 (18)	C5—C6—Rh1	70.8 (4)
C5—Rh1—Rh1^i^	102.03 (18)	C5—C6—C7	123.7 (6)
C5—Rh1—C2	82.0 (3)	C7—C6—Rh1	114.5 (4)
C5—Rh1—C6	38.3 (2)	C6—C7—C8	111.9 (5)
C6—Rh1—I1^i^	97.57 (17)	C1—C8—C7	112.7 (5)

Symmetry code: (i) −*x*+1, *y*, −*z*+1/2.

### Di-µ-iodido-bis{[(1,2,5,6-η)-cycloocta-1,4-diene]rhodium(I)} (P) Crystal data

**Table:**

[Rh_2_I_2_(C_8_H_12_)_2_]	*F*(000) = 1264
*M**_r_* = 675.97	*D*_x_ = 2.621 Mg m^−^^3^
Monoclinic, *P*2_1_/*c*	Mo *K*α radiation, λ = 0.71073 Å
*a* = 10.4505 (5) Å	Cell parameters from 9797 reflections
*b* = 19.390 (1) Å	θ = 2.6–26.4°
*c* = 8.6271 (4) Å	µ = 5.52 mm^−^^1^
β = 101.523 (2)°	*T* = 100 K
*V* = 1712.92 (14) Å^3^	Block, yellow-orange
*Z* = 4	0.14 × 0.14 × 0.09 mm

### Di-µ-iodido-bis{[(1,2,5,6-η)-cycloocta-1,4-diene]rhodium(I)} (P) Data collection

**Table:**

Nonius Kappa APEXII diffractometer	3519 independent reflections
Radiation source: sealed tube, fine-focus	2922 reflections with *I* > 2σ(*I*)
Graphite monochromator	*R*_int_ = 0.051
Detector resolution: 7.9 pixels mm^-1^	θ_max_ = 26.4°, θ_min_ = 2.0°
ω and φ scans	*h* = −13→13
Absorption correction: multi-scan (SADABS; Krause *et al.*, 2015)	*k* = −24→24
*T*_min_ = 0.057, *T*_max_ = 0.093	*l* = −9→10
38760 measured reflections	

### Di-µ-iodido-bis{[(1,2,5,6-η)-cycloocta-1,4-diene]rhodium(I)} (P) Refinement

**Table:**

Refinement on *F*^2^	Primary atom site location: dual
Least-squares matrix: full	Secondary atom site location: difference Fourier map
*R*[*F*^2^ > 2σ(*F*^2^)] = 0.025	Hydrogen site location: mixed
*wR*(*F*^2^) = 0.055	H atoms treated by a mixture of independent and constrained refinement
*S* = 1.07	*w* = 1/[σ^2^(*F*_o_^2^) + (0.0284*P*)^2^] where *P* = (*F*_o_^2^ + 2*F*_c_^2^)/3
3519 reflections	(Δ/σ)_max_ = 0.001
205 parameters	Δρ_max_ = 1.26 e Å^−^^3^
8 restraints	Δρ_min_ = −0.67 e Å^−^^3^

### Di-µ-iodido-bis{[(1,2,5,6-η)-cycloocta-1,4-diene]rhodium(I)} (P) Special details

**Table:**

Geometry. All esds (except the esd in the dihedral angle between two l.s. planes) are estimated using the full covariance matrix. The cell esds are taken into account individually in the estimation of esds in distances, angles and torsion angles; correlations between esds in cell parameters are only used when they are defined by crystal symmetry. An approximate (isotropic) treatment of cell esds is used for estimating esds involving l.s. planes.

### Di-µ-iodido-bis{[(1,2,5,6-η)-cycloocta-1,4-diene]rhodium(I)} (P) Fractional atomic coordinates and isotropic or equivalent isotropic displacement parameters (Å^2^)

**Table:**

	*x*	*y*	*z*	*U*_iso_*/*U*_eq_	
I1	0.73024 (3)	0.56233 (2)	0.46409 (3)	0.02018 (8)	
I2	0.89372 (2)	0.46580 (2)	0.19408 (3)	0.02188 (8)	
Rh1	0.80485 (3)	0.42940 (2)	0.45350 (4)	0.01765 (9)	
Rh2	0.72305 (3)	0.57016 (2)	0.14928 (4)	0.01730 (9)	
C1	0.7956 (4)	0.3230 (2)	0.3955 (5)	0.0205 (9)	
H1	0.778 (4)	0.318 (2)	0.280 (2)	0.025*	
C2	0.9215 (4)	0.3392 (2)	0.4781 (5)	0.0229 (9)	
H2	0.990 (3)	0.346 (2)	0.416 (4)	0.027*	
C3	0.9741 (4)	0.3226 (2)	0.6498 (5)	0.0253 (10)	
H3A	1.059815	0.345583	0.683485	0.030*	
H3B	0.988454	0.272270	0.660875	0.030*	
C4	0.8836 (4)	0.3453 (2)	0.7597 (5)	0.0261 (10)	
H4A	0.827646	0.306003	0.776878	0.031*	
H4B	0.937029	0.358549	0.863519	0.031*	
C5	0.7978 (4)	0.4056 (2)	0.6927 (5)	0.0241 (10)	
H5	0.814 (4)	0.4466 (15)	0.756 (5)	0.029*	
C6	0.6732 (4)	0.3985 (2)	0.5982 (5)	0.0198 (9)	
H6	0.607 (3)	0.4340 (16)	0.602 (5)	0.024*	
C7	0.6093 (4)	0.3303 (2)	0.5424 (5)	0.0234 (9)	
H7A	0.529071	0.339182	0.462128	0.028*	
H7B	0.583454	0.306526	0.632950	0.028*	
C8	0.6995 (4)	0.2832 (2)	0.4711 (5)	0.0240 (9)	
H8A	0.748312	0.253106	0.555256	0.029*	
H8B	0.645911	0.253237	0.390385	0.029*	
C9	0.6610 (4)	0.5469 (2)	−0.0943 (5)	0.0206 (9)	
H9	0.679 (4)	0.4967 (11)	−0.103 (5)	0.025*	
C10	0.7642 (4)	0.5945 (2)	−0.0773 (5)	0.0208 (9)	
H10	0.847 (3)	0.5720 (19)	−0.087 (5)	0.025*	
C11	0.7443 (4)	0.6700 (2)	−0.1207 (5)	0.0247 (10)	
H11A	0.665144	0.675101	−0.204767	0.030*	
H11B	0.819921	0.686915	−0.162912	0.030*	
C12	0.7293 (4)	0.7139 (2)	0.0242 (5)	0.0237 (9)	
H12A	0.816605	0.729810	0.079190	0.028*	
H12B	0.676206	0.755212	−0.012730	0.028*	
C13	0.6659 (4)	0.6751 (2)	0.1393 (5)	0.0228 (9)	
H13	0.685 (4)	0.693 (2)	0.250 (3)	0.027*	
C14	0.5529 (4)	0.6343 (2)	0.0998 (5)	0.0210 (9)	
H14	0.501 (3)	0.626 (2)	0.181 (4)	0.025*	
C15	0.4755 (4)	0.6267 (2)	−0.0676 (5)	0.0240 (9)	
H15A	0.485798	0.669082	−0.128031	0.029*	
H15B	0.381741	0.621703	−0.064695	0.029*	
C16	0.5200 (4)	0.5641 (2)	−0.1526 (5)	0.0233 (9)	
H16A	0.466146	0.523646	−0.136871	0.028*	
H16B	0.504985	0.573709	−0.267501	0.028*	

### Di-µ-iodido-bis{[(1,2,5,6-η)-cycloocta-1,4-diene]rhodium(I)} (P) Atomic displacement parameters (Å^2^)

**Table:**

	*U*^11^	*U*^22^	*U*^33^	*U*^12^	*U*^13^	*U*^23^
I1	0.02462 (15)	0.01759 (15)	0.01827 (15)	0.00106 (11)	0.00414 (11)	0.00015 (11)
I2	0.02106 (15)	0.02275 (16)	0.02346 (15)	0.00440 (11)	0.00840 (11)	0.00442 (12)
Rh1	0.01825 (17)	0.01641 (17)	0.01870 (17)	0.00044 (13)	0.00467 (13)	0.00062 (13)
Rh2	0.01825 (17)	0.01664 (17)	0.01712 (17)	0.00074 (13)	0.00379 (13)	0.00049 (13)
C1	0.028 (2)	0.014 (2)	0.021 (2)	0.0024 (17)	0.0100 (18)	−0.0021 (18)
C2	0.024 (2)	0.018 (2)	0.029 (2)	0.0044 (18)	0.0087 (18)	0.0019 (19)
C3	0.021 (2)	0.026 (2)	0.029 (2)	0.0061 (18)	0.0056 (18)	0.004 (2)
C4	0.032 (3)	0.022 (2)	0.023 (2)	0.0001 (19)	0.0030 (19)	−0.0015 (19)
C5	0.033 (3)	0.021 (2)	0.019 (2)	0.001 (2)	0.0093 (19)	0.0014 (19)
C6	0.019 (2)	0.019 (2)	0.022 (2)	0.0040 (17)	0.0069 (17)	−0.0012 (18)
C7	0.021 (2)	0.024 (2)	0.026 (2)	−0.0016 (18)	0.0056 (18)	0.0011 (19)
C8	0.028 (2)	0.020 (2)	0.024 (2)	0.0007 (18)	0.0052 (18)	−0.0011 (18)
C9	0.026 (2)	0.023 (2)	0.013 (2)	0.0013 (18)	0.0045 (17)	−0.0008 (18)
C10	0.023 (2)	0.025 (2)	0.018 (2)	0.0030 (18)	0.0099 (17)	−0.0014 (18)
C11	0.027 (2)	0.025 (2)	0.023 (2)	−0.0018 (19)	0.0065 (18)	0.0073 (19)
C12	0.029 (2)	0.018 (2)	0.022 (2)	−0.0015 (18)	0.0003 (18)	0.0016 (18)
C13	0.029 (2)	0.017 (2)	0.021 (2)	0.0051 (18)	−0.0001 (18)	−0.0013 (18)
C14	0.024 (2)	0.017 (2)	0.022 (2)	0.0081 (18)	0.0060 (18)	0.0019 (18)
C15	0.024 (2)	0.020 (2)	0.028 (2)	0.0031 (18)	0.0020 (19)	0.0023 (19)
C16	0.026 (2)	0.023 (2)	0.019 (2)	−0.0014 (18)	0.0011 (18)	−0.0002 (18)

### Di-µ-iodido-bis{[(1,2,5,6-η)-cycloocta-1,4-diene]rhodium(I)} (P) Geometric parameters (Å, º)

**Table:**

I1—Rh1	2.6998 (4)	C2—C3	1.509 (6)
I1—Rh2	2.7061 (4)	C3—C4	1.531 (6)
I2—Rh1	2.6833 (4)	C4—C5	1.516 (6)
I2—Rh2	2.6738 (4)	C5—C6	1.398 (6)
Rh1—C1	2.120 (4)	C6—C7	1.516 (6)
Rh1—C2	2.117 (4)	C7—C8	1.526 (5)
Rh1—C5	2.131 (4)	C9—C10	1.404 (6)
Rh1—C6	2.120 (4)	C9—C16	1.496 (6)
Rh2—C9	2.120 (4)	C10—C11	1.514 (6)
Rh2—C10	2.137 (4)	C11—C12	1.547 (6)
Rh2—C13	2.117 (4)	C12—C13	1.501 (6)
Rh2—C14	2.142 (4)	C13—C14	1.405 (6)
C1—C2	1.400 (6)	C14—C15	1.514 (6)
C1—C8	1.514 (6)	C15—C16	1.538 (6)
			
Rh1—I1—Rh2	88.274 (12)	C2—C1—Rh1	70.6 (2)
Rh2—I2—Rh1	89.290 (12)	C2—C1—C8	122.2 (4)
I2—Rh1—I1	85.839 (12)	C8—C1—Rh1	113.4 (3)
C1—Rh1—I1	159.56 (12)	C1—C2—Rh1	70.8 (2)
C1—Rh1—I2	93.80 (11)	C1—C2—C3	124.9 (4)
C1—Rh1—C5	90.54 (16)	C3—C2—Rh1	111.4 (3)
C2—Rh1—I1	161.67 (12)	C2—C3—C4	113.4 (3)
C2—Rh1—I2	90.72 (11)	C5—C4—C3	112.2 (3)
C2—Rh1—C1	38.60 (16)	C4—C5—Rh1	113.8 (3)
C2—Rh1—C5	81.67 (16)	C6—C5—Rh1	70.4 (2)
C2—Rh1—C6	97.81 (16)	C6—C5—C4	123.8 (4)
C5—Rh1—I1	96.17 (12)	C5—C6—Rh1	71.2 (2)
C5—Rh1—I2	161.76 (12)	C5—C6—C7	124.8 (4)
C6—Rh1—I1	91.38 (11)	C7—C6—Rh1	110.9 (3)
C6—Rh1—I2	159.83 (11)	C6—C7—C8	112.4 (3)
C6—Rh1—C1	81.93 (16)	C1—C8—C7	112.6 (3)
C6—Rh1—C5	38.41 (16)	C10—C9—Rh2	71.4 (2)
I2—Rh2—I1	85.902 (12)	C10—C9—C16	125.0 (4)
C9—Rh2—I1	157.60 (12)	C16—C9—Rh2	111.8 (3)
C9—Rh2—I2	92.63 (12)	C9—C10—Rh2	70.1 (2)
C9—Rh2—C10	38.52 (16)	C9—C10—C11	123.0 (4)
C9—Rh2—C14	81.27 (16)	C11—C10—Rh2	113.4 (3)
C10—Rh2—I1	163.81 (12)	C10—C11—C12	111.3 (3)
C10—Rh2—I2	92.82 (11)	C13—C12—C11	112.8 (3)
C10—Rh2—C14	89.99 (16)	C12—C13—Rh2	110.5 (3)
C13—Rh2—I1	92.62 (12)	C14—C13—Rh2	71.7 (2)
C13—Rh2—I2	155.19 (12)	C14—C13—C12	125.6 (4)
C13—Rh2—C9	97.80 (16)	C13—C14—Rh2	69.8 (2)
C13—Rh2—C10	81.78 (16)	C13—C14—C15	123.3 (4)
C13—Rh2—C14	38.53 (16)	C15—C14—Rh2	113.5 (3)
C14—Rh2—I1	95.06 (11)	C14—C15—C16	112.1 (3)
C14—Rh2—I2	166.28 (11)	C9—C16—C15	112.7 (3)
